# Weighted Gene Co-expression Network Analysis Identifies Specific Modules and Hub Genes Related to Subacute Ruminal Acidosis

**DOI:** 10.3389/fvets.2022.897714

**Published:** 2022-06-10

**Authors:** Qiuju Wang, Bingnan Gao, Xueqing Yue, Yizhe Cui, Juan J. Loor, Xiaoxia Dai, Xu Wei, Chuang Xu

**Affiliations:** ^1^College of Animal Science and Technology, Heilongjiang Bayi Agricultural University, Daqing, China; ^2^Key Laboratory of Low-Carbon Green Agriculture in Northeastern China, Ministry of Agriculture and Rural Affairs P. R. China, Heilongjiang Bayi Agricultural University, Daqing, China; ^3^Department of Animal Sciences, Division of Nutritional Sciences, University of Illinois, Urbana, IL, United States; ^4^The Royal Veterinary College, University of London, London, United Kingdom; ^5^Department of Biosystems, Division of Animal and Human Health Engineering, KU Leuven, Leuven, Belgium; ^6^Heilongjiang Provincial Key Laboratory of Prevention and Control of Bovine Diseases, Daqing, China

**Keywords:** subacute ruminal acidosis, bioinformatic analysis, differentially expressed genes, inflammatory response, transmembrane signaling

## Abstract

Weighted gene co-expression network analysis (WGCNA) was used to understand the pathogenesis of subacute ruminal acidosis (SARA) and identify potential genes related to the disease. Microarray data from dataset GSE143765, which included 22 cows with and nine cows without SARA, were downloaded from the NCBI Gene Expression Omnibus (GEO). Results of WGCNA identified highly correlated modules of sample genes, and Gene Ontology (GO) and Kyoto Encyclopedia of Genes and Genomes (KEGG) pathway enrichment analyses allowed further biological insights into SARA-related modules. The protein-protein interaction (PPI) network, modules from the PPI network, and cistron annotation enrichment of modules were also analyzed. A total of 14,590 DEGs were used for the WGCNA. Construction of a protein-protein network identified *DCXR, MMP15*, and *MMP17* as hub genes. Functional annotation showed that *DCXR* mainly exhibited L-xylulose reductase (NADP+) activity, glucose metabolic process, xylulose metabolic process, and carbonyl reductase (NADPH) activity, which are involved in the pentose and glucuronate interconversion pathways. *MMP15* and *MMP17* mainly have a collagen catabolic process. Overall, the results of this study aid the clarification of the biological and metabolic processes associated with SARA at the molecular level. The data highlight potential mechanisms for the future development of intervention strategies to reduce or alleviate the risk of SARA.

## Introduction

Subacute ruminal acidosis (SARA) is the most common nutritional disorder afflicting dairy cows. SARA occurs when the rumen pH is between 5.0 and 5.5 for a period of 3 h or more each day (1). The onset of SARA is associated with increased concentrations of short-chain fatty acids (SCFA), such as propionic acid, acetic acid, and butyric acid, created during feed fermentation. Particularly, the quantities of these SCFA exceed the absorption capacity of the rumen wall. Under these conditions, the large accumulation of SCFA in the rumen causes a gradual pH decrease. Hence, when SARA occurs, the chronic low pH environment causes a large number of microorganisms in the rumen to lyse and die, releasing a large amount of lipopolysaccharide (LPS), and damaging the rumen epithelial tissue (2, 3).

In intensive dairy production, the incidence of SARA ranges from 11 to 26%, and can reach >40% in some herds (4). According to estimates, the economic loss due to reduced milk production alone amounts to $400 per cow per one lactation (5, 6). Despite the grave economic losses caused by SARA in the international dairy industry, there is little scientific understanding of the pathophysiology that triggers SARA. Additionally, current research theories about using bioinformatics technology to explore the mechanism of SARA are lacking (7). The use of microarray technology in animal disease research has increased steadily, and bioinformatics data processing of gene expression profiles is widely used to discover potential new diagnostic and therapeutic targets that could help prevent disease (8).

In the past decades, SARA has been extensively studied, and some potential new therapeutic targets have been identified (9), including metabolic pathways of SCFA (10), immune cell suppression and inflammatory response (11), and lipocalin through the blood as preliminary diagnostic indicators of SARA (12). Particularly, the inflammatory response and the immune system have been recognized to have important effects on rumen epithelial health in lactating high-yielding dairy cows, in particular, those related to the systemic immune response associated with SARA (13, 14). Hence, it is important to better understand the genes and pathways that may be associated with systemic immunity and inflammation to further understand the pathophysiology of SARA (15).

Recent genome-wide association studies (GWAS), along with classical studies on metabolism in dairy cows, have increased our understanding of SARA. However, the molecular mechanisms underlying the regulation of the metabolic responses remain unclear (16). The weighted gene co-expression network analysis (WGCNA) algorithmic program clusters sequences into modules as a function of co-expression similarities across samples, leading to a cluster of genes with similar functions. The modules can then be related to one another, to whole-animal phenotypes, and help determine tissue-specific biomarkers and pathophysiological pathways (17, 18).

In the current study, we created correlation networks using publicly accessible resources based on the microarray dataset GSE143765 from the Gene Expression Omnibus (GEO). This study aimed to construct a sequence co-expression network to predict clusters of candidate genes concerned within pathological process associated with SARA. We screened for differentially expressed genes and constructed co-expression networks for all genes in the sample using WGCNA. Subsequently, cistron modules associated with SARA were identified. We used gene ontology (GO) and the genes and genomes (KEGG) pathway enrichment analyses to further uncover biological insights from modules that were highly correlated. Modules of protein-protein interaction (PPI) networks associated with SARA were also screened. In the present study, all potential genes were analyzed to ensure that our results were complete and reliable. The results of this study may facilitate elucidation of the pathophysiological features of SARA development at the molecular level. Potential molecular targets for the development of interventions to prevent or reduce the risk of SARA could be identified.

## Methods

### Collection of Microarray Data

The mRNA microarray expression profile dataset GSE143765 was downloaded from the gene expression omnibus (GEO) database. Data from the liver samples from 22 cows with SARA 9 cows without SARA were obtained from a study by Kizaki et al. (19) (https://www.ncbi.nlm.nih.gov/geo/query/acc.cgi?acc=GSE143765). The GEO database is a publicly accessible functional genomic database that contains high-throughput data, including microarrays. The GSE143765 dataset (GPL22092 [Agilent-072598]) was generated using the Bovine_cusotm8x15K platform (Agilent Probe version). We downloaded the raw TXT and the probe annotation files. The probes were assigned the corresponding gene symbols in steps using the annotation data on the platform. All information is freely available on the manufacturer's website. The present study did not include any experiments involving humans or animals.

### Data Preprocessing and DEG Screening

After downloading the raw data in TXT format, the Affymetrix package (20) (http://www.bioconductor.org/packages/release/bioc/html/affy.html) in R software (version 4.1.1, https://www.r-project.org/) was used for data preprocessing, and a robust multiarray average (RMA) was obtained after removing batch differences and performing data background correction, normalization, and summarization. To characterize DEGs, SARA and non-SARA groups were analyzed using the LIMMA (linear models for microarray data) package (21) within the R/Bioconductor platform. Benjamini–Hochberg's false discovery rate was used to reduce the likelihood of Type 2 errors and to adjust *P*-values < 0.05.

### Construction of Weighted Gene Co-expression Networks

As systems biology methodology, the development of cistron co-expression networks and the identification of cistron clusters or modules are particularly helpful in characterizing transcriptional alterations in multigene diseases. This is particularly true for phenotypes created by the convergence of small changes in gene expression rather than isolated single-gene effects (22, 23). The weighted factor co-expression network analysis (WGCNA) package (Version one.70-3, https://cran.rstudio.com/web/packages/WGCNA/index.html) in the R software package was used to construct groups of powerfully co-expressed genes into co-expression networks. WGCNA of all expressed genes (14,590 genes). The selection of soft-threshold power is a vital step during the construction of a WGCNA (24). Data preprocessing uses the WGCNA built-in goodSamplesGenes function to check missing items in gene data, items with weights below the threshold, and zero-variance genes. During module choice by cluster analysis, the contiguity matrix was used to calculate the topological overlap measure (TOM) and strength between all sequence pairs (25, 26). Modules of co-expressed genes were generated using stratified cluster dendrograms with completely different colors. The module structure was generated using topological overlap matrix plots. Finally, to ensure the reliability of the results of each module, the minimum number of genes in the module was set to five and the similarity module merging distance was 0.8. This allowed the identification of candidate genes with the highest correlation with SARA for further analysis and validation.

### Functional Enrichment Analysis of Genes in Key Modules of Cows With SARA

Gene modules with a *P*-value <0.05 and the highest correlation with SARA were selected. Analysis of cellular component (CC), molecular function (MF), and potential biological processes (BP) was performed to identify and interpret advanced biological functions supported by the GO terms and KEGG pathway annotation within the co-expression modules. Genes of every handpicked module were submitted to the web-based software DAVID (https://david.ncifcrf.gov/conversion.jsp) for practical and pathway enrichment analyses. DAVID is a helpful online tool for gene expansion (27–29), which provides the practicality of performing concurrent GO and KEGG pathway analyses. *P*-values <0.05 were used to uncover vital variations.

### Integration of Genetics and Highly Connected Hubs in Modules

The top-ranked genes within the modules were considered as hub genes. To consistently analyze hub genes in modules and module eigengenes, genes obtained from every module were mapped using the net search tool STRING information (30) (STRING, V11.5; https://string-db.org/), which can play a key role in identifying protein-protein networks (PPI). A combined score ≥0.4 of PPI pairs was defined as vital. The CytoHubba plugin-supported Cytoscape package (31) (http://www.cytoscape.org/version3.7.1; Institute for Systems Biology, Seattle, WA, USA) was used to construct and visualize the transcriptional regulatory network modules.

## Results

### Identification of DEGs Associated With Non-SARA and SARA Samples

Thirty-one raw tissue sample files (TXT format) were downloaded from the GEO information database. After batch normalization using the SVA and LIMMA packages in the R language, totally 15,068 probes on the comprehensive dataset GSE143765 were extracted with 14,646 gene expressions.

### Weighted Co-expression Network Construction

**Figure 1:**
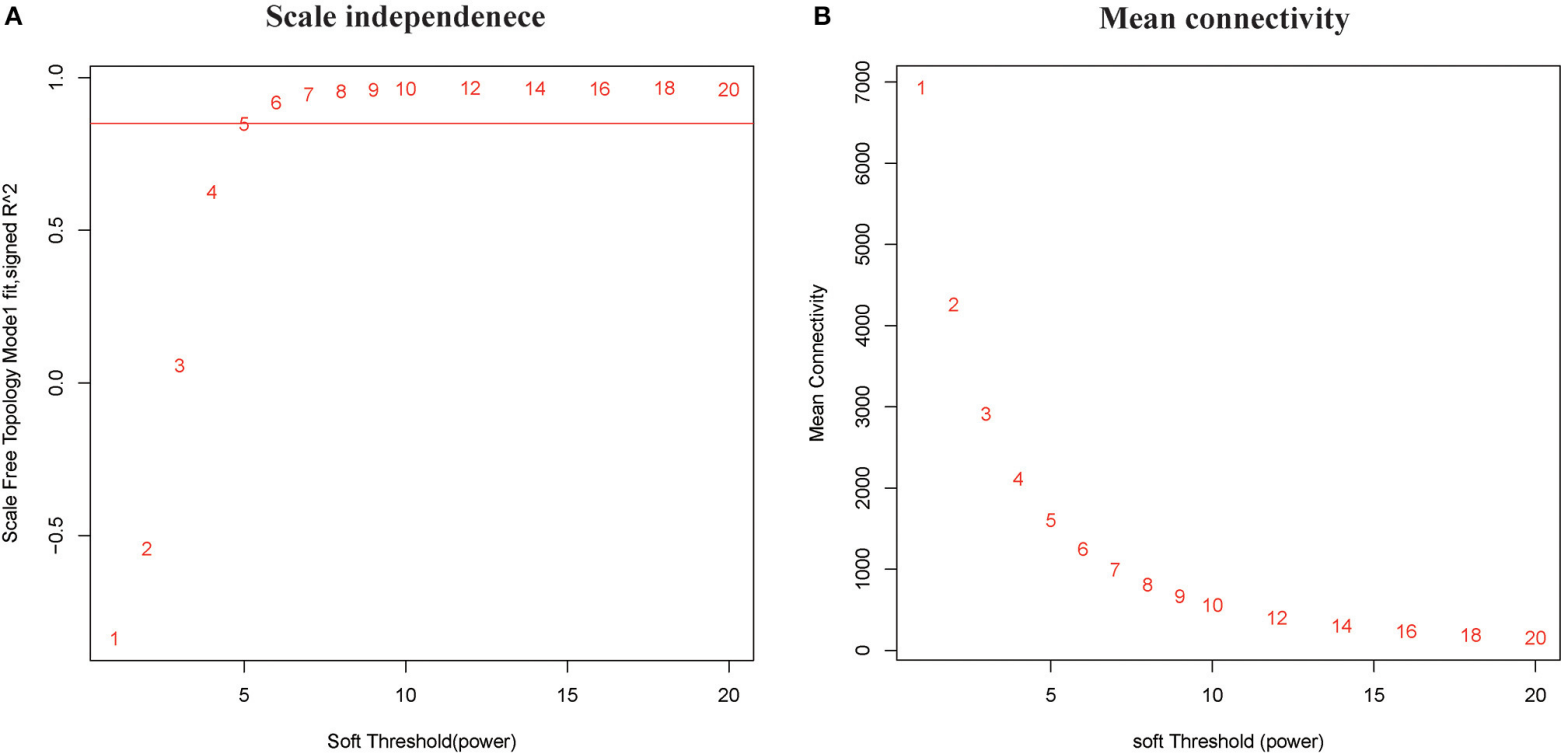
Clustering of samples and determination of soft-thresholding power. **(A)** Analysis of the scale-free fit index for various soft-thresholding powers (β). The red line represents the merging threshold. **(B)** Analysis of the mean connectivity for various soft-thresholding powers. In all, 7 was the most fit power value.

**Figure 2:**
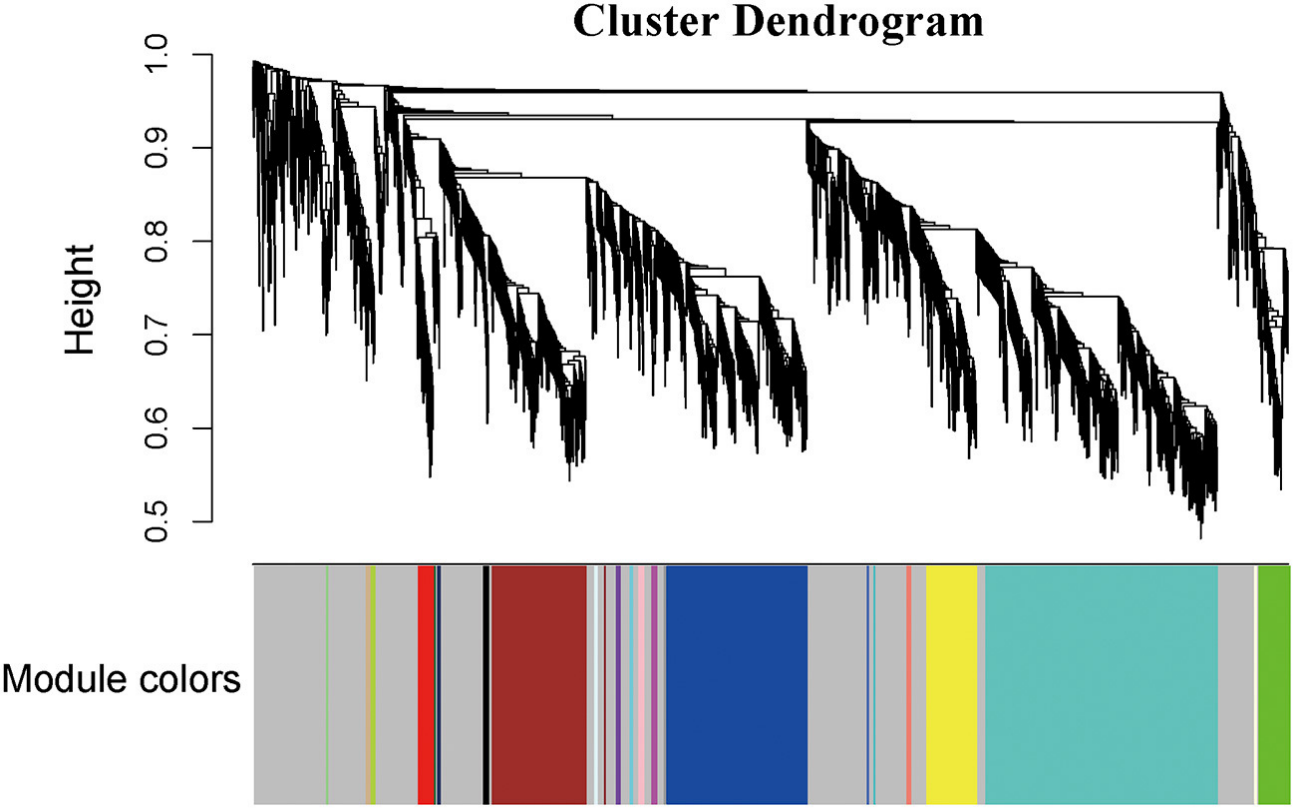
Construction of coexpression modules by the WGCNA package in R. The cluster dendrogram of genes in GSE143765. Branches of the cluster dendrogram of the most connected genes gave rise to 23 gene coexpression modules.Genes that could not be clustered into one of these modules were assigned to the gray module. Every gene represents a line in the hierarchical cluster. The distance between two genes is shown as the height on the *y*-axis.

In this study, soft threshold power was screened once the degree of scale independence was set to β = 7 (scale-free R2 = 0.95). When the degree of scale independence was set as β = 7, the measures decreased steeply with increasing soft threshold power, and subsequently, cistron co-expression module similarity and closeness matrices were obtained using the WGCNA algorithmic rule. After pretreatment, the number of genes was filtered from 14,590 to 2,464, we used WGCNA to spot the modules containing extremely correlated genes (Figures 1A,B). We set MEDissThres to 0.8 to merge similar modules, leading to 23 practical modules (Figure 2). There were 15 genes within the black module, 338 genes within the blue module, 228 genes within the brown module, 9 genes within the cyan module, 5 genes in dark green, dark red, dark-turquoise, and royal blue modules, 73 genes within the green module, 12 genes within the green-yellow module, 7 genes within the gray 60 module, 8 genes within the light-cyan module, 6 genes within the light-green module, 6 genes within the light-yellow module, 14 genes within the light-yellow module, 9 genes within the midnight-blue module, 15 genes within the pink module, 12 genes within the purple module, 38 genes within the red module,11 genes within the salmon module, 11 genes within the tan module, 558 genes within the turquoise module, and 122 genes within the yellow module. The 967 genes that may not be enclosed in any module were placed in the gray module, which was reserved for genes considered as not co-expressed.

### Correlation Between Modules and Identification of Key Modules

**Figure 3:**
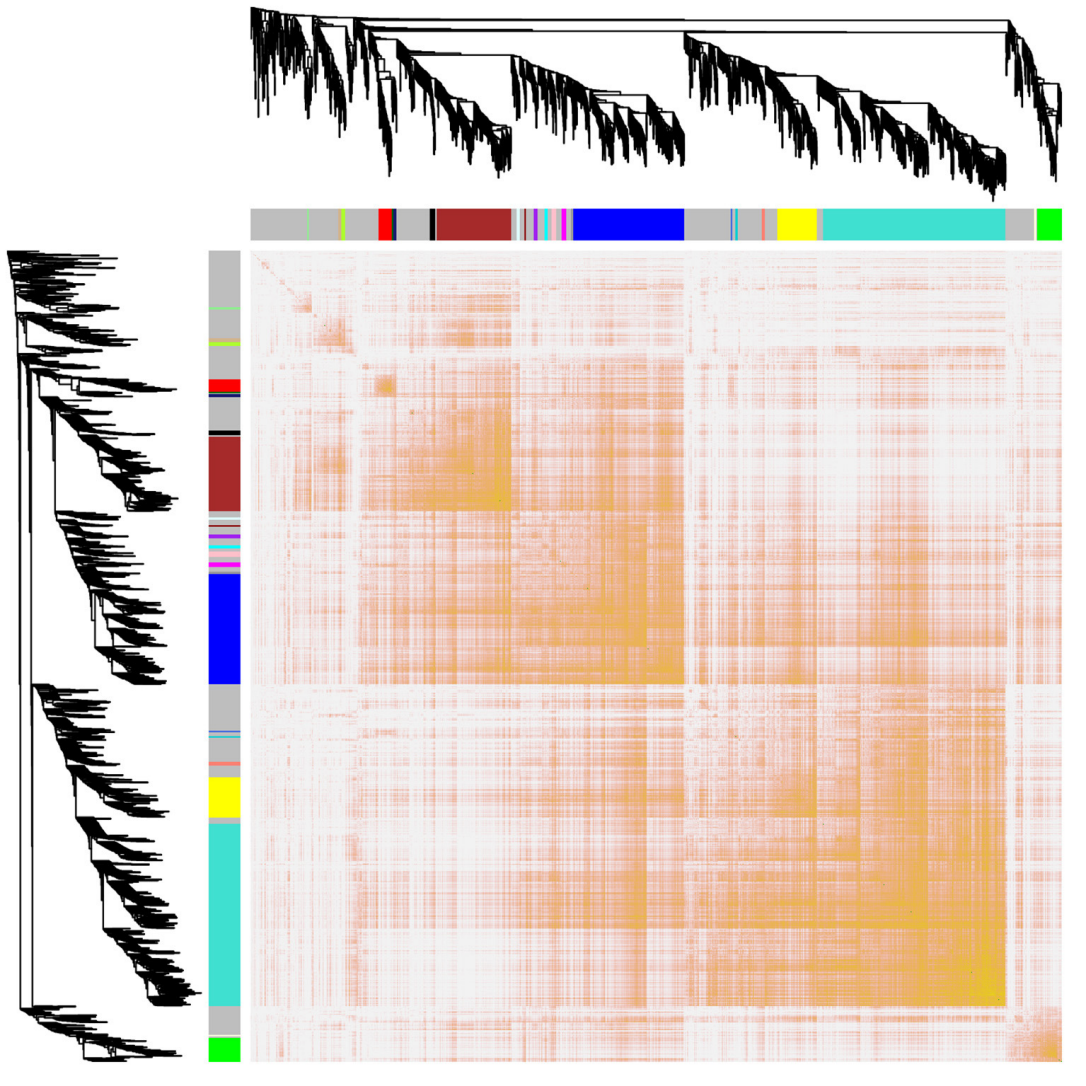
Interaction relationship analysis of coexpressed genes. Different colors of the horizontal axis and vertical axis represent different modules. The brightness of yellow in the middle represents the degree of connectivity of different modules. There was no significant difference in interactions among different modules, indicating a high degree of independence among these modules.

**Figure 4:**
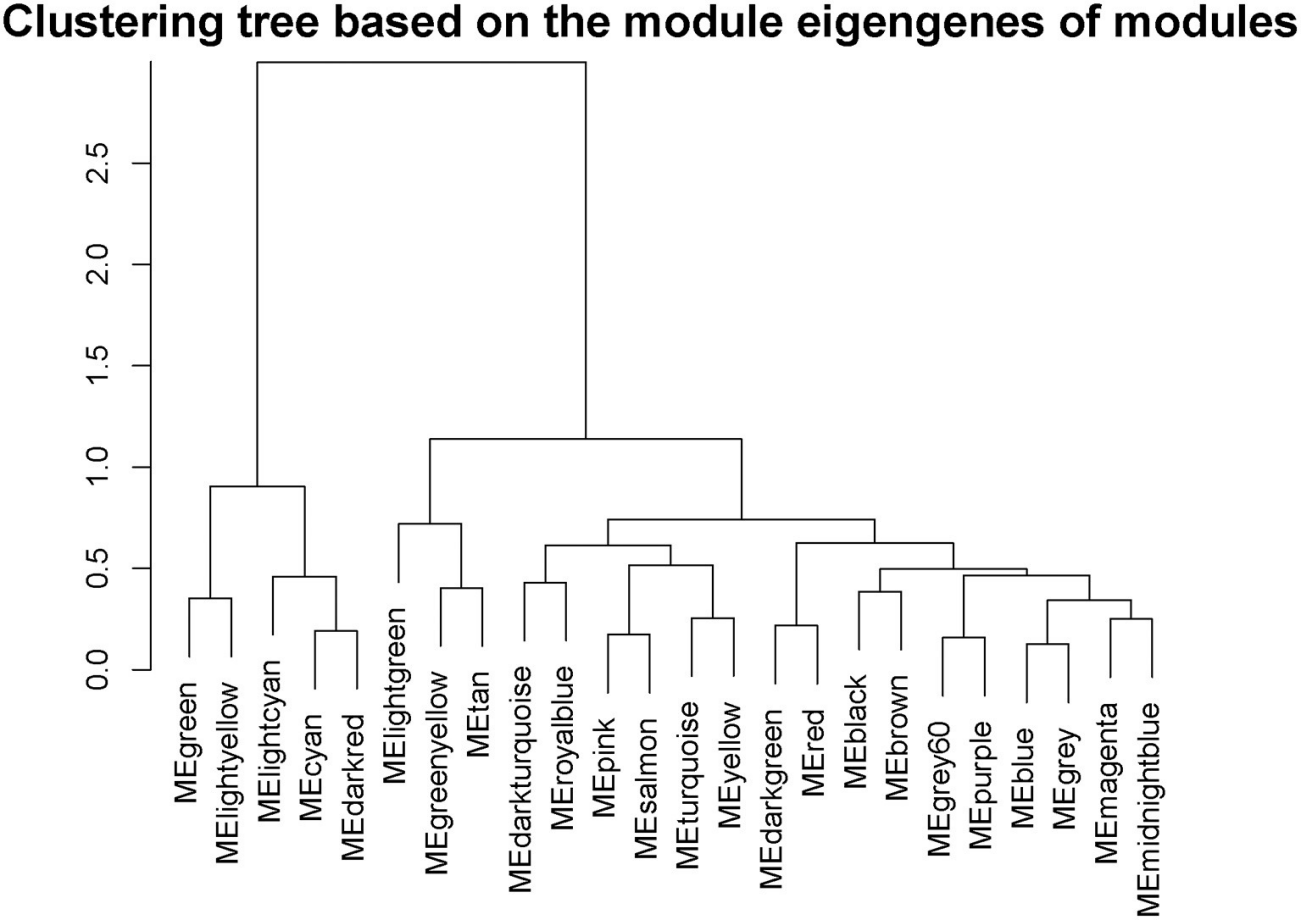
Hierarchical clustering of module hub genes that summarize the modules yielded in the clustering analysis.

**Figure 5:**
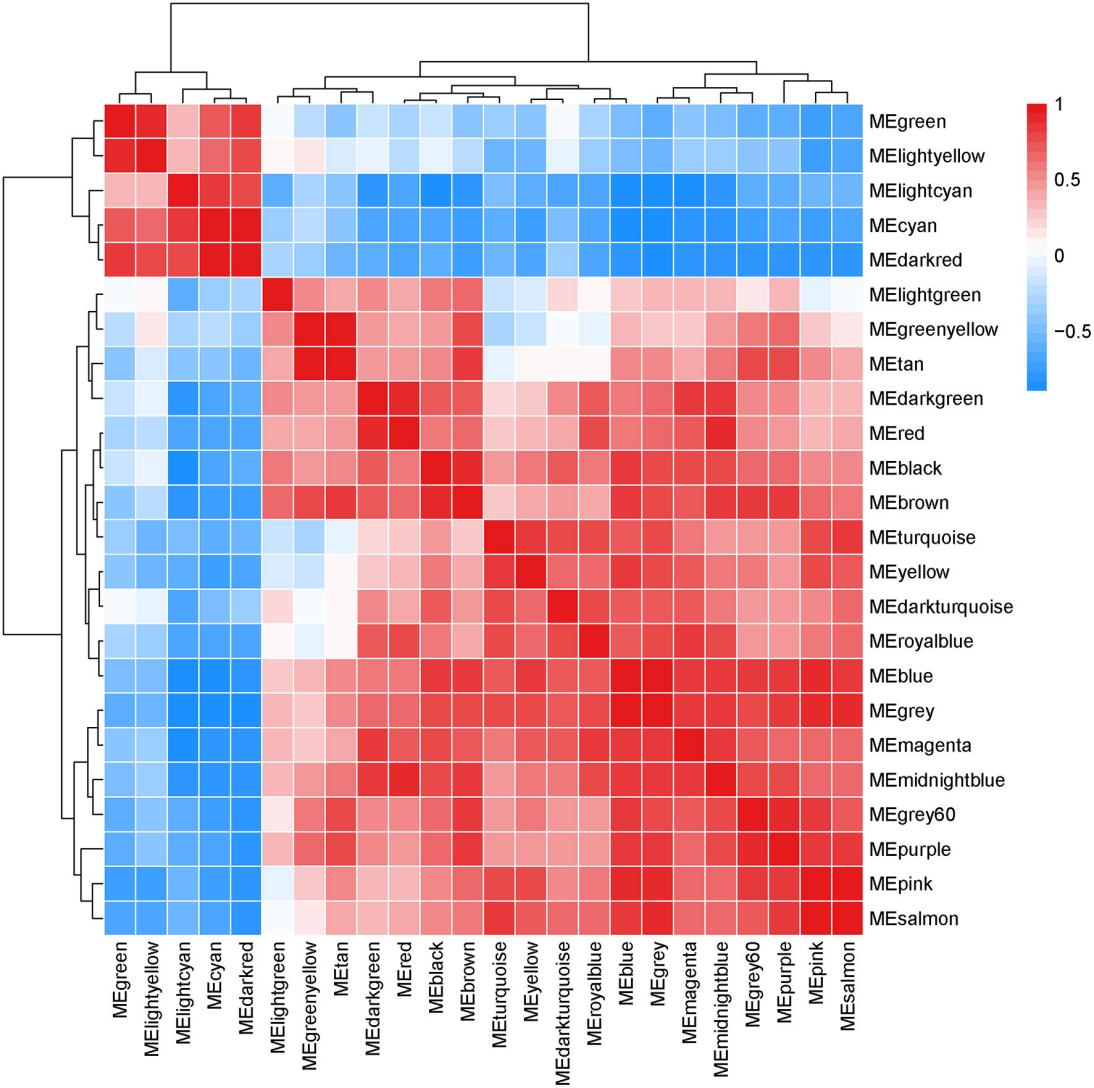
Heat map based on the results of C plots, with darker red being higher correlations.

**Figure 6:**
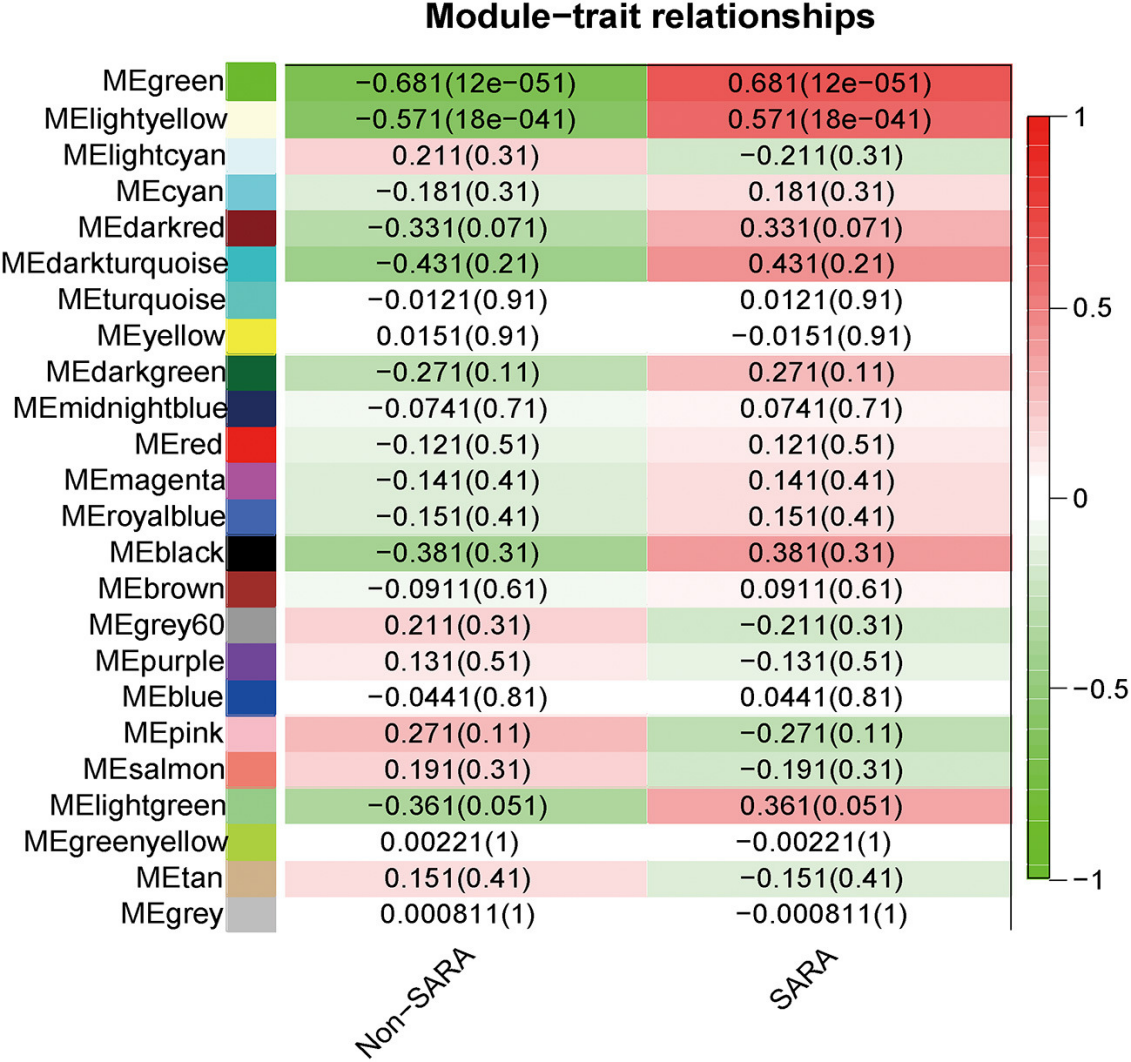
Correlation analysis of all modules with SARA samples and Non-SARA samples, positive correlations in red and negative correlations in green, values in parentheses are *p*-values, correlations were considered significant when *p*-values were < 0.05.

Interactions between the 23 co-expression modules were analyzed, and every gene is depicted within the network heatmap (Figure 3). Interestingly, the results revealed that a number of these sequence modules were valid, particularly, the light-green and inexperienced modules, with a high level of independence among the modules and relative independence of genes expressed in every module. To grasp the link between these 23 co-expression modules, we analyzed the genetic linkage of those 23 co-expression modules. Combined (Figure 4), we ascertained that these 23 modules were classified into two main clusters: one enclosed five modules (green, light yellow, light-cyan, cyan, and dark-red modules), whereas the other enclosed 18 modules (light-green, green-yellow, tan, dark-turquoise, royal-blue, pink, salmon, turquoise, yellow, dark-green, red, black, brown, grey60, purple, blue, magenta, and midnight-blue modules). Furthermore, the results were incontestable by the heatmap of eigengenes (Figure 5). We found that the proximity between modules in the two clusters was very high. The proximity between the cyan module and dark-red module was the highest in the five modules cluster, and the proximity between the tan module and green-yellow module, pink module, and royal-blue module was the highest in the cluster of 23 modules. The final correlation analysis and heat map plotting of the characteristic genes in all modules with the clinical information of SARA and non-SARA samples showed that (Figure 6) only the green and lightweight modules were significantly correlated with SARA (*P* < 0.05) and negatively correlated with non-SARA (*P* < 0.05).

### Functional Enrichment Analysis of Genes in the Module

**Figure 7:**
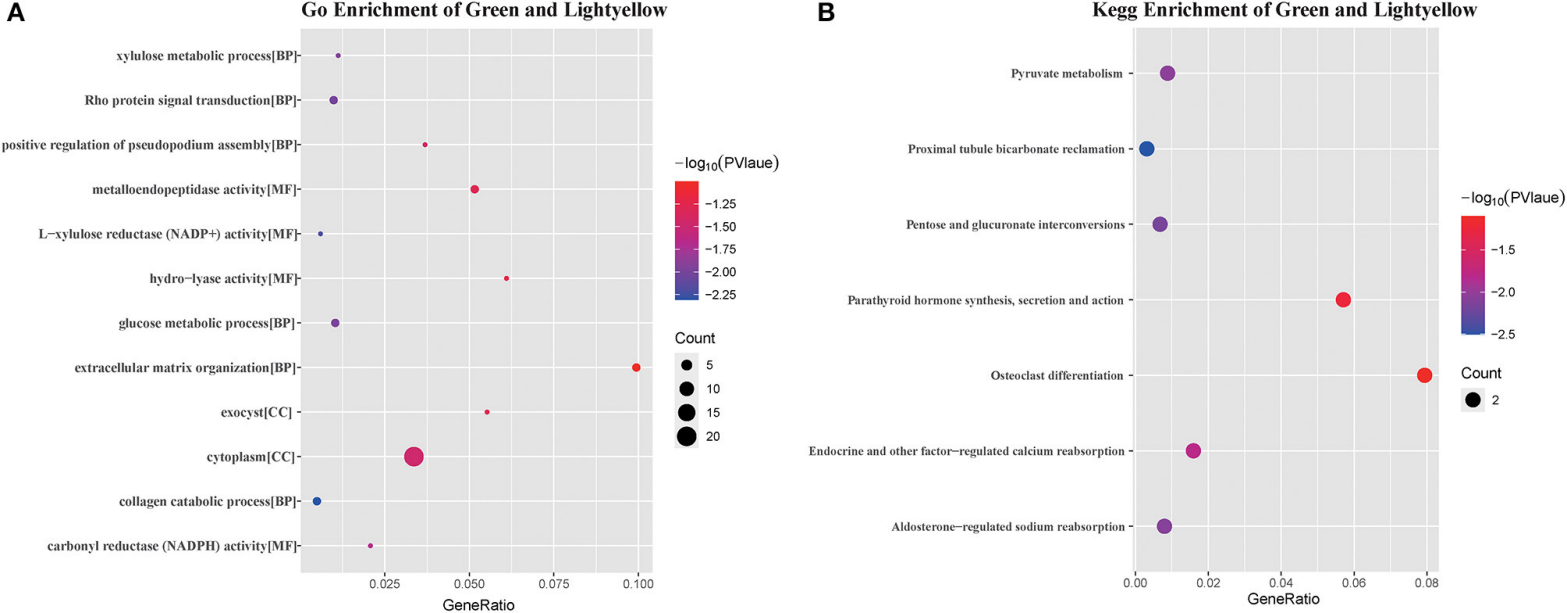
GO analysis and KEGG pathway enrichment results of genes in green and light yellow module. **(A)** GO enrichment results for the green and light yellow module, the bubble size shows the number of enriched GO terms containing genes from the green and light yellow module, the color represents the significance score of the enrichment, and the bubble nodes represent the ratio of the enriched genes to the GO terms. **(B)** KEGG enrichment of green and light yellow modules, bubble size shows the number of genes enriched to the biological pathway lexicon containing green and light yellow modules, color represents the color represents the significance score of enrichment, and bubble nodes represent the ratio of enriched genes to the KEGG lexicon.

As only the green and light-yellow modules were highly correlated with SARA, GO, and KEGG enrichment analyses were performed to explore the biological functions of the genes within the green and light-yellow modules. As shown in Figure 7A, genes in the green and light-yellow modules were mainly enriched in the cytoplasm to function with the molecular functions of L-xylulose reductase (NADP+) activity and carbonyl reductase (NADPH) activity, which are involved in the negative regulation of the collagen catabolic process, Rho protein signal transduction, glucose metabolic process, xylulose metabolic process, and positive regulation of pseudopodium assembly. KEGG pathway analysis (Figure 7B) showed proximal tubule bicarbonate reclamation, pentose and glucuronate interconversions, and aldosterone-regulated sodium reabsorption. Pyruvate metabolism and endocrine and other factor-regulated calcium reabsorption. These results showed that these genes were closely related to the nutritional metabolism and absorption functions such as carbohydrate, pyruvate, and calcium ion reabsorption.

### PPI Network Construction and Module Analysis

**Figure 8:**
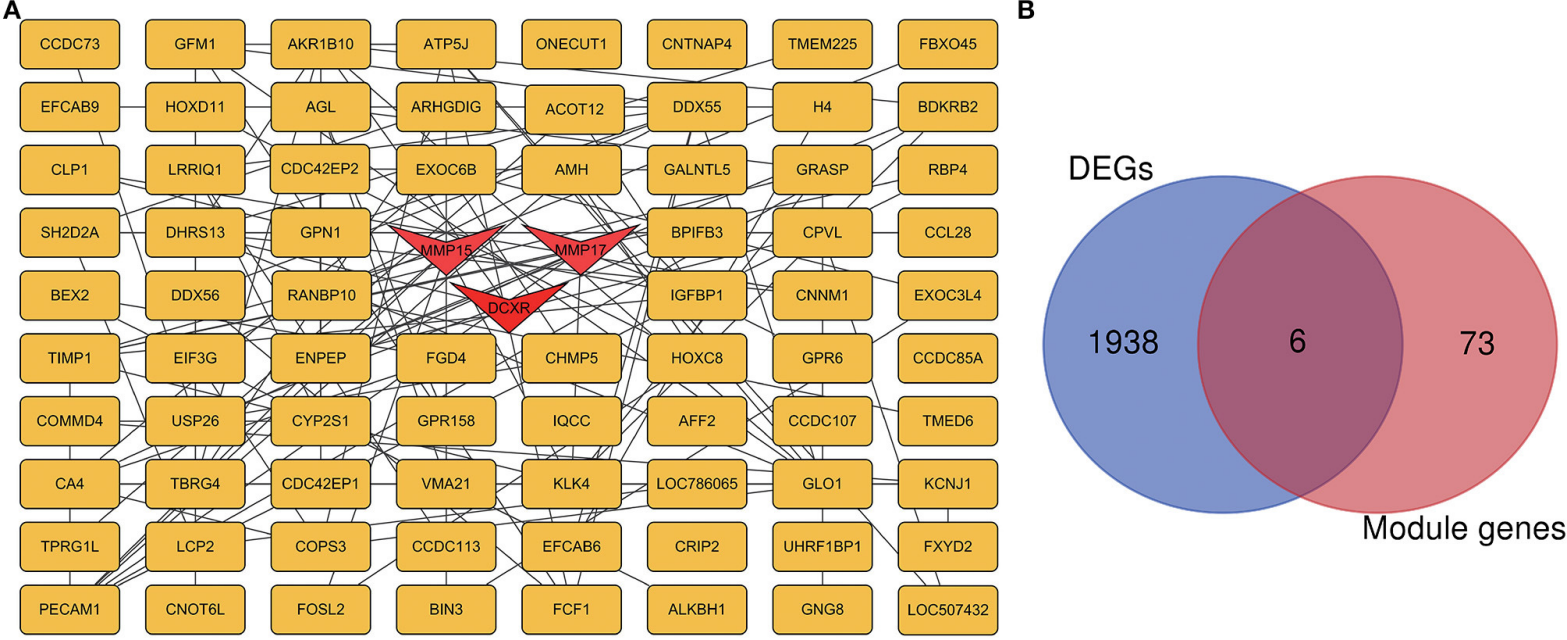
PPI network of hub genes in green and light yellow modules. **(A)** Green and light yellow module, with 79 nodes and 119 edges. Triangular nodes represent hub genes. **(B)** The blue circle is the differential gene, the red circle is the blue module and the light yellow module gene, and the common gene is the junction of the two circles.

The PPI network for all genes in the green and light yellow modules (Figure 8A) was constructed based on the STRING database and Cytoscape software. A high degree was calculated for the hub genes of the green and light-yellow modules using the cytoHubba plugin. As shown in Figure 8B, we crossed the genes in the green and light yellow modules with the differential genes and found that *TPRG1L, BDKRB2, CLP1, KLK4, RBP4*, and *DCXR* were the common genes. As shown in Table 1, we crossed genes in the green and light-yellow module with genes enriched in GO, and finally *MMP15, MMP17*, and *DCXR* genes were found common. Therefore, dicarbonyl and L-xylulose reductase (*DCXR*) is the crucial important HUB gene, because it has differential, modular, and HUB genes, whereas *MMP15* (matrix metallopeptidase 15) and *MMP17* (matrix metallopeptidase 17) have both modular genes and the HUB gene. *DCXR, MMP15*, and *MMP17* are enriched in biological processes and molecular functions, such as collagen catabolic process, L-xylulose reductase (NADP+) activity, glucose metabolism process, xylulose metabolism process, and carbonyl reductase (NADPH) activity.

**Table 1:** Most significant GO terms of the turquoise module.

**GO ID**	***P*-value**	**No. of genes**	**Description**	**Hub gene**
GO:0030574	0.004917312	3	Collagen catabolic process	MMP15 MMP17
GO:0050038	0.005971952	2	L-xylulose reductase (NADP+) activity	DCXR
GO:0007266	0.009858303	3	Rho protein signal transduction	N/A
GO:0006006	0.010339016	3	Glucose metabolic process	DCXR
GO:0005997	0.011214848	2	Xylulose metabolic process	DCXR
GO:0004090	0.020749112	2	Carbonyl reductase (NADPH) activity	DCXR
GO:0005737	0.033598223	20	Cytoplasm	N/A
GO:0031274	0.036903833	2	Positive regulation of pseudopodium assembly	N/A

## Discussion

Subacute ruminal acidosis (SARA) is a prevalent nutritional metabolic disorder in large-scale dairy farms (32, 33). Despite the progress made regarding the nutritional metabolism of SARA, further research is needed to investigate the relationship between SARA and the pathways related to immunosuppression and inflammatory responses. In recent years, microarray technology has become a popular technique that is often used to identify genes with altered expression during disease pathology (34). The GSE143765 dataset may be important for identifying SARA case physiology and biomarkers. Several researchers have previously analyzed this dataset, and the raw data of GSE143765 provided by Kizaki and Kim demonstrated that SARA differential genes in the liver were mainly enriched for genes involved in oxidation, suggesting that SARA is associated with high oxidative stress. Dong et al. divided sheep into a 40% low concentrate feed group and a 60% high concentrate feed group fed continuously for 9 weeks (35). The same was true for Abaker et al., who divided mid-lactation cows into 40% low and 60% high concentrate groups and found that the high concentration group developed SARA and had greater NF-kB gene expression and an inflammatory response, leading to increased oxidative stress (36). In the present study, WGCNA was used to analyze the molecular mechanisms of SARA samples compared to non-SARA samples. A total of 14,590 original genes were used to establish co-expression networks and to identify high co-expression gene sets. Moreover, 23 different co-expression modules were identified based on the genes processed by the original gene, and the functions of the modules were analyzed based on the co-expression network to obtain the modules most related to SARA (green and light yellow modules).

Over the past 20 years, research has largely established that when SARA occurs in cows, metabolic disorders, inflammatory responses, and immunosuppression primarily occur (37). Many studies have shown that compared with the non-SARA group, a large amount of VFA is produced in the rumen of dairy cows in the SARA group. When VFA is higher than the limit of VFA absorption in the rumen, the pH value in the rumen is reduced to damage the rumen barrier, affect the absorption of nutrients in the rumen, and finally accumulate in the liver, causing nutritional metabolic regulation disorders (38). The green and light-yellow module genes were mainly involved in nutritional metabolism. The enrichment of glucose and xylose in the sugar metabolism is due to the fact that when the lactating cows have SARA symptoms, the energy provided by the feed cannot be absorbed due to the damage of the rumen. Therefore, the liver mobilizes the decomposition of adipose tissue and reduces insulin sensitivity to provide energy for the body (39). The reason for the enrichment of L-xylulose reductase (NADP+) and carbonyl reductase (NADPH) activities is that NADPH is the main component in the process of obtaining energy in dairy cows (40). The collagen catabolic process, Rho protein signal transduction, and positive regulation of pseudopodium assembly are mainly enriched because LPS is produced in the rumen tissue when SARA occurs in dairy cows, and liver damage and systemic inflammation are produced by blood circulation (41). The genes in the green and light-yellow modules were mainly enriched in functions related to carbohydrate metabolism, energy metabolism, and immune regulation, which indicates that the genes in the green and light-yellow modules are involved in the nutritional metabolism and inflammatory response process in dairy cows suffering from SARA diseases. Through the analysis of the PPI network, differential gene screening, and functional enrichment of genes in the green and light-yellow modules, the genes with intersection of the two and above were identified as *DCXR*, and *MMP15* and *MMP1*7 were the key genes. Many studies have shown that *DCXR* plays a crucial role in fat and glucose metabolism. Palombo et al. also used DCXR as a candidate marker gene for fat metabolism in genome-wide association studies of milk fatty acid composition in Italian Holstein cows (42). M*MP15* and *MMP17*, as HUB genes, are involved in embryonic development, reproduction, and tissue damage recovery during normal physiological processes, however, they promote inflammation, such as arthritis and fibrosis, when the disease occurs. (43), Therefore, although the results of *MMP17* and *MMP15* are low, it is not difficult to find that both of them increase gene expression after SARA disease, hence, they should be involved in the inflammatory response and damage repair of the rumen and liver tissues (44).

Pathway enrichment analysis showed that genes in green and light-yellow modules were enriched in proximal tubule bicarbonate reclamation, pentose and glucuronate interconversions, aldosterone-regulated sodium reabsorption, pyruvate metabolism, and endocrine and other factor-regulated calcium reabsorption pathways. The inflammatory response is typically mediated by the release of large amounts of inflammatory mediators, activation of inflammatory cells such as monocytes and macrophages, and release of pro-inflammatory mediators, such as TNF-α and interleukin-1B (IL-1B) (45). In recent years, it has been found that the SARA-induced inflammatory response mainly occurs in rumen tissue, mainly because when suffering from SARA, the pH value in the rumen decreases, leading to bacterial fragmentation, releasing a large amount of LPS to damage the rumen tissue barrier, which eventually leads to LPS translocation to the peripheral blood circulation (46). Because the liver is an important organ of blood circulation, translocated LPS and LPS binding protein (LBP) eventually enter the liver, leading to liver disease (47). However, in recent years, in-depth studies on the inflammatory response of SARA have found that when dairy cows suffer from SARA, stress is induced and calcium, non-esterified fatty acids, and glucose concentrations are reduced, and glucocorticoid receptor protein in the liver is significantly downregulated, which is consistent with the enrichment results of this experiment (48). Although LPS translocation eventually leads to liver inflammation, liver inflammation after a series of reactions has rarely been explored.

This study had certain limitations. First, this study focused on two aspects of data mining and data analysis, both of which are genetic methodologies, and the results were not validated experimentally. Second, the samples were obtained from peripartal Holstein cow liver samples; hence, only the liver gene expression analysis during SARA was performed and association analysis with blood samples was not performed. Therefore, the results of this study should be understood within the context of its limitations.

## Conclusions

Our study adopted a systems biology-based WGCNA methodology and uncovered various helpful molecular targets for future investigations of the mechanisms and choice of SARA biomarkers. Some essential biological processes and pathways, collagen catabolic process, L-xylulose reductase (NADP+) activity, glucose metabolism, xylulose metabolism, NADPH activity, pentose and glucuronate interconversions, and other pathways, as well as hub genes that play a role in these processes, may help to elucidate the process of SARA production and development. Moreover, potential molecular mechanisms include Rho protein signal transduction, positive regulation of pseudopodium assembly and proximal tubule bicarbonate reclamation, aldosterone-regulated sodium reabsorption, pyruvate metabolism, and endocrine and other factor-regulated calcium reabsorption signaling pathways. Our findings may facilitate the development of new experiments to better understand the pathophysiology and progression of SARA. Further studies should be conducted to validate the value of the resulting genes in the context of SARA.

## Data Availability Statement

The original contributions presented in the study are included in the article/supplementary material, further inquiries can be directed to the corresponding author/s.

## Author Contributions

QW, BG, and CX conceived the study. QW, BG, and XY participated in method development and validation. BG and YC carried out the data analysis. QW and BG prepared the original draft. JL, XD, and XW reviewed and modified the manuscript. All the authors have read and approved the final version of the manuscript.

## Funding

This work was supported by Major Science and Technology Project of Heilongjiang Province (Grant No. 2021ZX12B03), the Provincial Institute Cooperation Project of the Heilongjiang Science and Technology Plan (Grant Nos. YS20B04 and YS19B01), the Development Project of Local Universities in Heilongjiang Bayi Agricultural University (ZRCQC201803 and ZRCLG201904), the Postdoctoral Scientific Research Developmental Fund of Heilongjiang Province (LBH-Q20161), and the National Natural Science Foundation of China, Beijing, China (Grant Nos. 32125038 and 32072931).

## Conflict of Interest

The authors declare that the research was conducted in the absence of any commercial or financial relationships that could be construed as a potential conflict of interest.

## Publisher's Note

All claims expressed in this article are solely those of the authors and do not necessarily represent those of their affiliated organizations, or those of the publisher, the editors and the reviewers. Any product that may be evaluated in this article, or claim that may be made by its manufacturer, is not guaranteed or endorsed by the publisher.

## Abbreviations

SARA: subacute ruminal acidosis
GO: Gene Ontology
KEGG: Kyoto Encyclopedia of Genes and Genomes
BP: biological process
MF: molecular function
CC, cellular component. PPI: protein-protein interaction.
